# Hepatocellular carcinoma in Senegal: epidemiological, clinical and etiological aspects about 229 cases at *Hôpital Principal de Dakar*

**DOI:** 10.11604/pamj.2021.38.99.25195

**Published:** 2021-01-28

**Authors:** Ibrahima Diallo, Bineta Ndiaye, Mouhamed Touré, Abdoul Sow, Ababacar Mbengue, Papa Silman Diawara, Sara Boury Gning, Papa Saliou Mbaye, Fatou Fall, Mouhamadou Mbengue

**Affiliations:** 1 Department of Internal Medicine and Hepatogastroenterology, Hôpital Principal de Dakar, Dakar, Senegal,; 2 Department of Medical Biology, Hôpital Principal de Dakar, Dakar, Senegal,; 3 Department of Medical Imaging, Hôpital Principal de Dakar, Dakar, Senegal,; 4 Department of Internal Medicine and Hepatogastroenterology, Hôpital Général de Grand-Yoff, Dakar, Senegal

**Keywords:** Hepatocellular carcinoma, hepatitis B virus, Senegal

## Abstract

Hepatocellular carcinoma (HCC) is a major public health problem in Senegal, and the third most common cancer in terms of incidence. However, there are no recent data on the characteristics of this pathology in our country. The aim was to describe the epidemiological, clinical, aetiological and therapeutic aspects of HCC at Hôpital Principal de Dakar, Senegal. We did a descriptive retrospective study, including patients hospitalized from January 2012 to December 2017. We included 229 patients. The mean age was 47.4 years (21 - 88 years), and 77 patients (33.6%) were under 40 years of age. The sex ratio was 6.6. Twelve patients (5.2%) had a family history of 1^st^ degree cirrhosis or HCC. Ten patients (4.4%) were previously treated with nucleotide analogues. The most common clinical sign at diagnosis was abdominal pain (91.7%). Alpha-fetoprotein level was normal in 12.2% of patients, and greater than 400 ng/ml in 68.1% of cases. Abdominal ultrasound found nodular HCC in 122 patients (68.2%), infiltrative HCC in 19 patients (10.6%), and was normal in 38 cases (21.2%). Subjacent cirrhosis was detected in 71.3% of cases. Abdominal computed tomography (CT) scan showed compatible HCC lesions in 88.8% of cases. A histological diagnosis was obtained in 2 patients (0.9%). The most common etiological factor was hepatitis B virus (69.4%), characterized mostly by a younger age (p = 0.001). In 20.9% of cases, no aetiology was found. An advanced or terminal stage (BCLC C/D) was found in 217 cases (94.8%). The treatment was curative in 12 patients (5.2%), and palliative in 7 cases (3.1%). The evolution at one year was favourable in 6 patients (2.6%). Hepatocellular carcinoma (HCC) is a disease that mainly affects young male adults in Senegal. The main aetiological factor remains HBV infection. The diagnosis is made at an advanced stage and the prognosis very bad.

## Introduction

Hepatocellular carcinoma (HCC) is the most common primary liver tumor. It is the sixth cancer in the world by incidence. In 2018, 841,000 new cases of primary liver cancer were registered worldwide, including 75 to 85% of HCC [1]. In Senegal, HCC represents a real public health problem. According to GLOBOCAN 2018 data, it is the second leading cause of cancer in men and the third in women in our country [1]. The main risk factors of HCC are hepatitis B (HBV) and hepatitis C viruses (HCV), non-alcoholic steatohepatitis (NASH), alcohol, aflatoxin, hemochromatosis, diabetes, male sex and smoking [2]. Hepatocellular carcinoma is a tumour with a very poor prognosis particularly in resource-poor countries where the annual fatality ratio is around 96% [3]. Its management depends on the stage at diagnosis, most often assessed by the classification of the Barcelona Clinic Liver Cancer (BCLC). In developed countries the diagnosis is usually done early, but in sub-Saharan Africa, it is often made when the disease is at an advanced stage. The aim of our study is to describe the epidemiological, clinical, aetiological and therapeutic aspects of HCC at Hôpital Principal of Dakar in Senegal.

## Methods

This is a retrospective and descriptive study including patients hospitalized in the departments of internal medicine and hepato-gastroenterology at Hôpital Principal of Dakar from January 1^st^ 2012 to December 31^st^ 2017, for HCC. The diagnosis of HCC was based on one of the following three criteria: histological evidence of HCC, or the combination of 2 morphological aspects of HCC (typical vascular hallmarks of HCC on ultrasound, CT-scan or magnetic resonance imaging (MRI) (hypervascularity in the arterial phase with washout in the portal venous or delayed phase)), or a morphological criterion associated with an increase in the level of alpha fetoprotein (AFP) greater than 400 ng/ml. The etiological diagnosis of HCC was retained for HBV when at least one of the markers (HBsAg, anti-Hbc antibodies, HBV DNA) was presented: for HCV if HCV antibodies or HCV ribonucleic acid (RNA) was presented, for alcohol when consumption has been regular for at least 10 years even if it was not quantified, and for autoimmune hepatitis when autoantibodies (anti-nuclear Ab, anti-LKM1 Ab, anti-smooth muscle Ab) were positive. The other contributing factors were considered to be a probable aetiology of HCC if all the examinations of the previous diagnosis were negative. We did not include patients with another primary or secondary liver tumour, as well as those with an unusable or incomplete medical record. The informations were collected using an anonymous operating sheet. The data collected were epidemiological (age, sex), clinical (discovery circumstances, general and functional signs, data from the clinical examination), biological (hepatic functional explorations, alphafetoprotein, hemogram, calcemia, etc.), radiological (ultrasound, CT, MRI), histological, therapeutic (curative, palliative or symptomatic), and outcomes. Records were analysed with Epi info software version 7.1.

## Results

We included 229 patients with HCC, representing a hospital prevalence of 0.37%, and a prevalence in hepato-gastroenterology services of 3.5%. The average age of the patients was 47.4 (21 - 88) years. Among our patients, 77 (33.6%) were under 40 years of age, and 57 (24.9%) were between 40 and 49 years of age. The male/female ratio was 6.6, with 199 men (86.9%) and 30 women (13.1%). The average age was the same by gender. Among our patients, 9 (3.9%) reported chronic carrier of HBV in the family, and 12 (5.2%) reported a history of chronic liver disease (cirrhosis or cancer) in a first-degree relative. Ten patients (4.4%) were on treatment with nucleot(s)ide analogs (lamivudine in one patient and tenofovir diproxil fumarate in the others) for chronic carriage of HBV at the time of diagnosis (6 patients with cirrhosis and 4 patients with active chronic hepatitis). Regular alcohol consumption was found in 22 patients (9.6%). The other antecedents are summarized in Table 1. Hepatocellular carcinoma was diagnosed as part of a semi-annual screening in 12 (5.2%) patients (10 patients with chronic HBV on treatment, 1 for autoimmune cirrhosis and 1 for alcoholic cirrhosis).

**Table 1:** baseline characteristics of the patients

	Number of patients	Percentage
Familial chronic HBV	9	3.9
First degree family story of liver disease (cirrhosis or HCC)	12	5.2
Treated for chronic HBV	10	4.4
Diabetes mellitus	19	8.3
Alcohol	22	9.6
Smoking	54	23.6
Alcoholic cirrhosis	1	0.4
Autoimmune cirrhosis	1	0.4
Overweight / Obesity	11	4.8
**Performance status**		
WHO 0	13	5.7
WHO 1	46	20.1
WHO 2	59	25.7
WHO 3	78	34.1
WHO 4	33	14.4
**Symptoms**		
Abdominal pain	210	91.7
Hepatomegaly	170	74.2
Jaundice	144	62.9
Oedema	96	41.9
Ascites	76	33.2
Collateral venous circulation	37	16.2
Splenomegaly	29	12.7
Hepatic encephalopathy	11	4.8
**Risk factors (n= 229)**		
HBV	159	69.4
HBV only	122	53.3
HBV + HCV	2	0.9
HBV + HIV	2	0.9
HBV + Alcohol	13	5.7
HBV + Diabetes mellitus	11	4.8
HBV + Overweight	9	3.9
HCV	3	1.3
Alcohol	9	3.9
Autoimmune disease	1	0.4
Overweight / Obesity	9	3.9
No risk factor found (incomplete investigations)	48	20.9

At diagnosis, the performance status of patients was good (0 or 1) in 59 patients (25.8%), moderate in 59 patients (25.7%) and bad (3 or 4) in 111 cases (48.5%) (Table 1). The clinical signs were dominated by abdominal pain which was found in 210 patients (91.7%), hepatomegaly in 170 cases (75.9%) and jaundice in 144 cases (64%). Signs of portal hypertension were found in 142 patients (62%). The other signs are reported in Table 1. Liver functions evaluation showed that 213 patients (93%) had cytolysis, cholestasis was present in 89 patients (38.9%), and 36 patients (15.7%) had isolated hyperbilirubinemia. Data for Child-Pugh classification were available for 171 patients with Child-Pugh A in 27 cases (15.8%), Child-Pugh B in 102 patients (59.6%) and Child-Pugh C in 42 cases (24.6%) (Table 2). The others biological characteristics were hypercalcemia in 35 patients (15.3%), anemia in 105 patients (45.9%) and thrombocytopenia in 43 cases (18.8%) (Table 2). Alpha fetoprotein testing was performed in all patients. The normal threshold was 12 ng/ml. The dosage was often qualitative beyond 2000 ng/ml. Twenty-eight patients (12.2%) had a normal AFP level, 44 (19.2%) had a rate between 12 and 400ng/ml, and 157 (68.6%) had a value greater than 400 ng/ml (Table 2).

**Table 2:** biological characteristics and Child-Pugh classification

Characteristics (n patients)	Number of cases/ Number of patients	Percentage
Hepatic cytolysis (n= 213)	213/229	93
<2N	37	17.4
2- 5N	83	39
>5N	93	43.6
Cholestasis (n= 229)	89	38.9
Isolated hyperbilirubinemia (n= 229)	36	15.7
**Prothrombin time (n= 220)**		
<50	41	18.6
50-70	91	41.4
>70	88	40
**Albumin (n= 171)**		
<28	81	47.4
28-35	49	28.6
>35	41	24
**AFP (ng/ml) (n= 229)**		
1-12	28	12.2
12-400	45	19.6
400-105000	156	68.1
Child-Pugh classification (n= 171)		
Child-Pugh A	27	15.8
Child-Pugh B	102	59.6
Child-Pugh C	42	24.6

Data from abdominal ultrasound were collected in 179 patients (Table 3). They showed nodular lesions of the liver compatible with HCC in 122 patients (68.2%). The average size of the nodular lesions was 6.5 cm (1.2 to 17 cm). An infiltrative form of HCC was found in 19 patients (10.6%). Among patients with HCC, 91 (64.5%) had dysmorphia of the non-tumor liver, and 50 (35.5%) had the rest of the liver parenchyma morphologically normal. Abdominal ultrasound didn´t find a lesion compatible with HCC in 38 cases (21.2%). Associated lesions were portal thrombosis in 73 patients (40.8%), and ascites in 80 cases (44.7%). CT scan was performed in 134 patients, 84 of whom had an ultrasound which didn´t find a tumour lesion in 29 cases. It showed lesions compatible with HCC in 119 cases (88.8%) (Table 3). MRI was performed in 12 patients, and confirmed HCC diagnosis in 11 patients (91.7%). Liver biopsy was performed in two cases (0.9%), and confirmed the diagnosis in these patients. In some patients with extension assessment, imaging found pulmonary metastases in 20 cases (8.7%), spinal and bone metastases in 4 cases (1.7%) as well as peritoneal carcinosis in 4 patients (1.7%).

**Table 3:** radiological findings

Characteristics (n patients)	Number of cases/ Number of patients	Percentage
**Abdominal ultrasonography (n= 179)**		
Nodular HCC	122	68.2
1 nodular lesion	59	33
2 nodular lesions	13	7.3
3 nodular lesions	3	1.7
More than 3 nodular lesions	47	26.2
Infiltrative HCC	19	10.6
No suspicious lesion of HCC	38	21.2
Hepatic dysmorphia	36	20.1
Normal	2	1.1
**Aspect of non tumoral parenchyma (n= 141)**		
Cirrhotic	91	64.5
Normal	50	35.5
Portal vein thrombosis	73	40.8
Ascites	80	44.7
**CT scan (n= 134)**		
Lesions of HCC	119	88.8
Liver cirrhosis with portal vein thrombosis without HCC lesion	7	5.2
Non-specific focal lesion	4	3
Aspect of secondary liver tumor	3	2.2
Normal	1	0.7

Upper gastrointestinal endoscopy, performed in 108 patients (47.1%), was normal in 54 patients (50%), and found grade I oesophageal varices in 16 cases (14.8%), grade II in 26 cases (24.1%), grade III in 13 cases (12%), gastric varices in 8 patients (7.4%) and portal hypertensive gastropathy in 11 cases (10.2%). In summary after the biological and morphological examinations, the diagnostic strategy for HCC in our patients was a high level of alpha-fetoprotein greater than 400 ng/ml (68.6%) associated with abdominal ultrasound in 95 cases (41.5%), abdominal CT scan in 55 cases (24%) or MRI scan in 7 cases (3.1%). In patients with an alpha-fetoprotein level below 400 ng/ml (31.4%), the diagnosis of HCC was based on ultrasound with MRI and/or CT scan in 44 cases (19.2%), a CT scan associated with MRI in 2 patients (0.9%) and a CT scan alone in 24 patients (10.5%). The diagnosis was done by histology in 2 patients (0.9%). The most common cause of HCC was HBV infection. It was found in 159 patients (69.4%). This infection was isolated in 122 cases (53.3%), associated with HCV infection in 2 patients (0.9%), with HIV in 2 cases (0.9%), with alcohol consumption in 13 cases (5.7%), overweight or obesity in 9 cases (3.9%) or type 2 diabetes in 11 cases (4.8%). The other causes of HCC were an isolated HCV infection in 3 patients (1.3%), alcohol in 9 cases (3.9%), and autoimmune cause in 1 patient (0.8%). Seven (3.1%) and 2 patients (0.9%) had only diabetes mellitus and obesity with a BMI greater than 30 kg/m^2^ as potential risk factors but they didn´t have liver biopsy to confirm or not NAFLD/NASH. In 48 patients (20.9%), no risk factor was found. However, it should be noted that the investigations were not exhaustive in these cases.

The comparative study of the group of patients with HBV risk factor and the group of those with HCC of undetermined aetiology showed a mean age of 42.6 years in the first group versus 58.4 years in the second group (p = 0.001), a sex ratio of 7.4 and 5.7 respectively, a normal AFP rate in 9.4% and 19.4% (p = 0.01), and a non-tumoral liver morphologically normal in 23, 6% vs 9.5% (p = 0.001). According to the BCLC staging, 227 patients (94.8%) were in advanced or terminal stage (Table 4). A curative treatment was done in 12 patients (5.2%), and a palliative treatment performed in 7 patients (3.1%) which consisted of chemoembolization in each case. The other patients received symptomatic treatment which varied between tramadol and oral morphine. The outcome was favourable in 6 patients (2.6%) with a survival of at least one year: 4 after hepatectomy (current survival of 2, 3 and 5 years), 1 after radiofrequency (current survival of 4 years), 1 after percutaneous alcohol injection (current survival of 3 years). Eighty-seven patients (38%) died during hospitalization, 90 (39.3%) within 3 months of diagnosis, 13 (5.7%) after 3 months but within a year and 33 (14.4 %) patients were lost to follow-up.

**Table 4:** staging and treatment

Characteristics (n patients)	Number of cases/ Number of patients	Percentage
**BCLC staging (n= 229)**		
BCLC A	8	3.5
BCLC B	4	1.8
BCLC C	99	43.2
BCLC D	118	51.5
**Treatment**		
Curative treatment	12	5.2
Hepatectomy	6	
Destruction by radio frequency	2	
Percutaneous alcohol injection	4	
Palliative treatment	7	3.1
**Chemoembolization**		
Symptomatic treatment	210	91.7

## Discussion

Hepatocellular carcinoma (HCC) is a real public health problem in Senegal, which is located in the endemic area of HBV. However, there are no recent data on the characteristics of this pathology in our country. In the absence of a national cancer registry, we carried out a study in our hospital which is one of the reference structures in the country with one of the largest digestive diseases department, to have data that approximate the general population. According to our hospital cancer registry from January 2001 to December 2014, HCC represented 13.2% of all cancers hospitalized during this period and 30.4% of digestive cancers. Thus, we report retrospective data of 229 patients over 6 years, i.e. a hospital prevalence of HCC of 0.37%. However, the incidence of HCC has decreased in our country, going from 30/100,000 inhabitants in 1981 [4], to 12.6/100,000 inhabitants in 2018 according to the results of GLOBOCAN [1]. However, these statistics do not really reflect the reality in our regions, because of under-medicalization, many cases are not diagnosed or reported.

Hepatocellular carcinoma in Senegal is a disease of young adults with a mean age of 47.4 years, and 33.6% of patients with less than 40 years. These patients are older than in the study reported in 1981 where the mean age was 38 years [4]. Our data are substantially similar to the results of the various studies conducted in sub-Saharan Africa. Thus, a multicenter study, conducted in Nigeria, Ghana, Ivory Coast, Tanzania, Malawi and Sudan and concerning 1552 cases of HCC found a mean age of 45 years, with a proportion of 40% of patients who were under 40 years [5]. HCC in sub-Saharan Africa is a disease of young adults, unlike North Africa, Japan, Western Europe and North America, where the average age is between 52 and 69 years [5-11]. This is due to the fact that these regions represent areas of high HBV endemicity and that infection most often occurs at a very young age. In Senegal, chronic HBV is the first risk factor of HCC, and this infection occurs early between 0 and 5 years of age by perinatal and horizontal transmission. Even though practices have been improved, a few years ago, many deliveries were done at home and screening for HBV infection during pregnancy was not systematic. Furthermore, the close contact of young children with chronic carriers within the family or in the community is also an important factor of early transmission in our country.

There was also a clear male predominance in our study with a sex-ratio of 6.6. This confirms the male predominance in patients with HCC in Senegal. The GLOBOCAN results in 2018 found an incidence of 18 and 8.4 per 100,000 inhabitants respectively among men and women [1]. This male predominance is also reported in the other series, but with proportions varying between 1.3 in the Central African Republic and 6.14 in China [5-9,11-18]. A family history of liver disease (cirrhosis or cancer), a risk factor for developing HCC, was found in 12 patients (5.2%). In two case-control studies, the Odd-Ratio for occurrence of HCC when there was a family history of first-degree liver cancer was 4.1 (95% CI, 1.3-12.9) in United States [19], and 2.38 (95% CI, 1.01-5.58) in Italy [20]. Thus, the existence of a family history of HCC should lead to earlier and more regular surveillance, particularly in chronic HBV carriers. Hepatocellular carcinoma in Senegal is also characterized by a diagnosis at a late stage. Thus, a painful hepatomegaly is present in 75 to 90% of patients, as in the series reported in sub-Saharan Africa where it is reported in 69 to 100% of cases [12-14], and anomalies of liver function tests with high rate of cytolysis was found in 95.1% of cases, and reflects the advanced stage of the disease and hepatocellular insufficiency. In our patients, the diagnosis was made through screening in only 5.2% of cases. This rate is relatively low compared with studies in the developed countries where the diagnosis is often made during screening. In this case the rate of asymptomatic patients can exceed 50% at the time of diagnosis [9], hence the advantage of monitoring the patients at risk.

This advanced stage of the disease is also reflected by imaging. The mean diameter of the nodular lesions in our patients was 6.5 cm, and the abdominal ultrasound was sufficient in 78.8% of cases to make the diagnosis. In sub-Saharan Africa, the diagnosis of HCC is late, and most studies have reported an average tumour size at diagnosis greater than 6 cm [5,21,22], unlike studies in developed countries where the average size lesions ranged from 2.5 to 3.8cm [7]. Portal vein thrombosis, an element of diagnostic orientation, but also of advanced stage of HCC, was found in 40.8% of our patients, whereas it was only present in 10 to 29% of cases in studies in the developed countries [7-9,17]. Thus, ultrasound, with its availability and ease of performance, retains its place in the diagnosis of HCC in sub-Saharan Africa. When abdominal ultrasound was not able to diagnose HCC in our patients, CT-scan and MRI were useful. In 27/29 patients who had had a normal abdominal ultrasound, the CT scan made it possible to correct the diagnosis. The operator-dependent nature of the ultrasound could explain the fact that our patients had a normal examination when the scanner subsequently found a tumour lesion. MRI, due to its limited availability in our country, is mainly reserved for doubtful cases and for the characterization of small lesions. The liver biopsy was only performed in two cases in our patients, also reflecting the delayed diagnosis. On the other hand, it made it possible to make the diagnosis of small lesions, without elevation of AFP, in 15%, 21.4% and 63% of the cases respectively in Brazil, Italy and Germany [8,9,17]. However, in view of the performance of imaging for the diagnosis of HCC, the place of biopsy is currently considerably reduced.

Alpha-fetoprotein is also an important element for the diagnosis of HCC in our country, and is available in most health facilities. A high rate greater than 400ng/ml, combined with imaging, allowed us to retain the diagnosis in 68.6% of our patients. However, it was normal in 12.2% of our patients, while this rate varied between 15.5% and 48% of cases in studies in developed countries [8,9]. Thus, even if according to EASL the sensitivity of AFP for screening for HCC varies between 39 and 65%, and its use not recommended in diagnostic strategies [23], it currently retains its interest in sub-Saharan Africa, due to the limited availability of CT-scan and MRI in many localities. The most common cause of HCC in our patients was HBV carriage with 69.4% of cases. Unfortunately, viral load and genotyping have not been performed in our patients because of the advanced stage of the liver disease. Our data are in compliance with data in sub-Saharan Africa which is an area of very high prevalence of HBV, which is responsible for 55 to 75% of cases of HCC [5,13,24]. HBV infection occurs in more than 90% of cases in early childhood due to horizontal and perinatal transmission [25,26]. Thus, HCC occurs at a younger age (42.8 years) compared to those who were not infected with this virus. The male predominance is also more marked with a sex ratio of 8.35. Thus, male patients infected with the hepatitis B virus should be particularly monitored to avoid progression to HCC. On the other hand, the seroprevalence of HCV in our patients is very low (1.5%). Senegal is a country of low HCV prevalence, with a rate varying between 1 and 3%.

In our series, apart from tumour lesion, a cirrhotic liver was found in 72.2% of cases, and 27.8% of the patients had the remainder of the liver parenchyma which was morphologically normal. This is in compliance with the data from studies realized in sub-Saharan Africa which show that HCC develops in 60 to 70% of cases in liver cirrhosis [5,14]. In North Africa, Europe and Japan, HCC occurring on a cirrhotic liver is reported in 81 to 98% of cases [5,6,8,15,17,18,27]. This difference could be explained in part by the importance of HCV infection or alcoholic liver disease in the occurrence of HCC in these countries, while in sub-Saharan Africa their roles are less. In our study, 22 patients regularly drank alcohol, and of these, 13 had HBV infection. As a result, we only retained the isolated alcoholic aetiology in 9 patients (3.9%). On the other hand, the role of alcohol in the occurrence of HCC is 17.6% in the Maghreb [6], varies between 18 and 52% in Europe and North America [7-9,18], and from 4% to 13% in Asia [7]. In our patients with diabetes mellitus (3.1%) or overweight (0.9%) as the only etiological factor, the assertion of HCC secondary to NASH could not be established. Indeed, an exhaustive etiological investigation eliminating metabolic or autoimmune causes has not been realized. There was also a significant proportion of HCC without risk factor found in our study (20.9%). Identical rates are found in The Gambia with 18%, around 20% in the sub-Saharan countries in the study of Yang *et al*. and 12% in Egypt in the same study [5,14]. In developed countries, a risk factor is found in all studies in patients with HCC. This reflects a lack of exhaustive etiological investigation in sub-Saharan Africa due to the lack of resources, but also to the fact that at the time of diagnosis, the disease is so advanced that only the viral aetiologies are sought due to the costs. In addition, in our country where there is a high consumption of peanuts, the search for aflatoxin is not common in practice and had not been carried out in our patients.

The BCLC classification of our patients showed that 94.7% of them were at an advanced (C) or terminal (D) stage. The same proportions are found in the multicentric study of Yang *et al*. where only 5% of patients from sub-Saharan Africa were at BCLC A or B stage [5]. These results reflect a late diagnosis in our regions, and contrast with Maghreb, European, American or Asian data, where the diagnosis is made at an early stage, with BCLC 0 and A stages varying from 7 to 50% [7-9,18,28]. This also reflects the absence of a screening program in our countries, even though half of our patients who received curative treatment were screened as part of regular ultrasound monitoring. In view of the late diagnosis and the advanced stage of the disease, only 8.3% of our patients received specific treatment, either for curative or palliative purposes. These data are similar to the results of studies in sub-Saharan Africa where less than one percent of patients received specific treatment [5]. In Western and Asian studies, due to earlier screening, curative treatment rates between 34 and 65% are reported [7,8,18]. In our study, 2.6% of our patients had a favourable outcome. This is in agreement with the poor prognosis of HCC which is described in the literature. One-year survival was 1.1% in sub-Saharan Africa in the study of Yang *et al*. [5], while it exceeds 50% in Western countries where it can reach more than 10% at 5 years [7,29]. And if the diagnosis of HCC is made early as part of a screening, the survival rate can go up to 60% at 5 years as it is the case in Japan [10].

## Conclusion

Hepatocellular carcinoma is a condition that mainly affects middle-aged adults, with a clear predominance among men in Senegal. It evolves silently and is only diagnosed when it is at an advanced stage. The main risk factor remains HBV infection. Due to the late diagnosis, only a small number of patients receive specific treatment. As a result, HCC has a very poor prognosis with high mortality. Improving awareness of HBV vaccination, screening for chronic HBV infection, and implementation of HCC screening program for patients at risk, would make possible to prevent this pathology or to be able to treat it early in our country.

### What is known about this topic

- Hepatocellular carcinoma is a public health problem in sub-Saharan Africa;
- The disease is often found at an advanced stage;
- Hepatitis viruses are the leading cause of hepatocellular carcinoma in sub-Saharan Africa. The prognosis is very bad.

### What this study adds

- This study confirms the late diagnosis of hepatocellular carcinoma;
- It specifies the major role of the hepatitis B virus in the occurrence of hepatocellular carcinoma in Senegal, which gives hope for the reduction of this scourge with vaccination against HBV;
- It also shows that early detection is possible in sub-Saharan Africa as well as curative treatment.
